# 586. Characterizing the Clinical Outcomes of Patients with Positive Blood Cultures Not Identified by Rapid Diagnostics

**DOI:** 10.1093/ofid/ofad500.655

**Published:** 2023-11-27

**Authors:** Caleb C McLeod, Karen K Tan, Jacinda Abdul-Mutkabbir

**Affiliations:** Santa Clara Valley Medical Center, San Jose , California; Loma Linda University Medical Center, Loma Linda, California; University of California San Diego, La Jolla, California

## Abstract

**Background:**

Rapid diagnostics tools (RDTs) have improved clinical outcomes by prompt identification of pathogens in blood cultures, leading to the initiation of directed empiric antibiotic therapy. However, several organisms are unidentified by RDTs, which may delay appropriate antimicrobial therapy and increase mortality risks. Studies characterizing these off-panel organisms are limited, and guidance for antimicrobial therapy is unavailable. We sought to characterize the off-panel organisms encountered in blood cultures and provide important insights into the pathogens that may be missed by RDTs to improve the clinical management of bacteremia.

**Methods:**

This was a retrospective study conducted from 8/2018 to 5/2021 at an academic medical center in Loma Linda, California. All adult patients with blood cultures processed on the Verigene (RDT) platform were screened for inclusion. Patients were included if Verigene was unable to detect a target organism. Species identification and antimicrobial susceptibility were performed with MicroScan WalkAway or sent to a reference laboratory if unidentifiable by MicroScan. Patient demographics, comorbidities, suspected source of infection, discharge disposition, and readmissions were determined by chart review.

**Results:**

Out of 1,071 blood cultures screened, 80 (47 Gram-positive [GP] and 33 Gram-negative [GN] isolates were not identified by Verigene. While not significant, patients with off-panel GN bacteremia were older (65.5 vs 58 years, p=0.074) and had longer durations of stay (11 vs 9 days, p=0.149) than patients with unidentified GP isolates. Patients with off-panel GN isolates were also more likely to have a comorbid malignancy than those with off-panel GP isolates (42% vs 26%, p=0.112) and more likely to present with severe sepsis (89% vs 79%, p =0.376), though these differences were not significant. Cefepime and piperacillin/tazobactam were reliable agents for GN isolates (11/13, 18/21), and vancomycin retained activity against unidentified GP isolates.

**Conclusion:**

Patients with GN organisms not identified by RDTs are more likely to present with severe sepsis and less likely to survive hospital discharge than those with unidentified GP organisms. Further research is needed to guide antimicrobial therapy for off-panel results.

**Disclosures:**

**Jacinda Abdul-Mutkabbir, PharmD,MPH**, Entasis Therapeutics: Advisor/Consultant|Entasis Therapeutics: Honoraria|shionogi: Advisor/Consultant|shionogi: Honoraria

